# Psychological Effects of the Allocation Process in Human–Robot Interaction – A Model for Research on *ad hoc* Task Allocation

**DOI:** 10.3389/fpsyg.2020.564672

**Published:** 2020-09-18

**Authors:** Alina Tausch, Annette Kluge, Lars Adolph

**Affiliations:** ^1^Department Products and Work Systems, Federal Institute for Occupational Safety and Health, Dortmund, Germany; ^2^Chair of Work and Organisational Psychology, Ruhr University Bochum, Bochum, Germany

**Keywords:** work psychology, HRI, theory, participation, decision perception, satisfaction, self-organizing sociotechnical system

## Abstract

Task allocation is immensely important when it comes to designing human–robot interaction (HRI), but although it is the shaping part of the interaction, it is merely regarded as a process with its own effects on human thinking and behavior. This study aims at linking research from different fields like psychological theory, HRI and allocation optimization to create a new process model of *ad hoc* task allocation in human–robot interaction. It addresses the process characteristics and psychological outcomes of a real-time allocation process that integrates the worker. To achieve this, we structured the process into steps and identified relevant psychological constructs associated with them. The model is a first step toward ergonomic research on the self-organized allocation of tasks in HRI, but may also be an inspiration for practitioners designing HRI systems. To create successful work in HRI, designing the technology is an important foundation, but a participative, thought-out process for allotting tasks could be the key to adequate autonomy, work satisfaction and successful cooperation.

## Introduction

Human–robot interaction (HRI) has the potential to change the way of working in production permanently. The possibilities offered by the use of direct cooperation between humans and robots are far-reaching: especially for productions of low batch sizes with high variance, where full automation is not flexible enough, a hybrid production setting can be a solution for increasing efficiency (Chen et al., 2014). HRI offers the opportunity to design production in a flexible and adaptable manner, and to allow for low-threshold changes thanks to easier programming. It can also relieve workers ergonomically from monotonous or physically exhausting tasks and can enrich work through a complex interaction with technology. To fully unlock these potentials and to not risk a devaluation of work, a well thought-out allocation of tasks, defined as the distribution of tasks between humans and machines as part of work organization (Older et al., 1997), is of vital importance. HRI is highly dependent on humans and robots working together. In order to create synergies, the design of an appropriate allocation process that leads to acceptance instead of reactance is crucial (Older et al., 1997). This article therefore takes a human-centered perspective on task allocation in HRI and proposes a model to describe *ad hoc* allocation processes – integrated into the work process and relying on self-organization – and their psychological effects.

What is currently needed is a view on synergies of HRI and human-centered design that goes beyond the focus of task allocation research on decision criteria. The idea to develop algorithms that calculate optimal or near-to-optimal solutions of allocation problems shapes the literature (see e.g., Gerkey and Mataric, 2016), but new questions arise that cannot be answered with MABA–MABA (“men are better at, machines are better at”) principles. Some of these questions involve deciding which specific human should execute a task or if there are opportunities for an allocation that is adaptable by workers (see e.g., Older et al., 1997) and how this could be implemented. If one loses sight of human needs in the planning of allocation and focuses too much on technology, this can have adverse effects: Focusing only on “perfect” algorithms and fully automating allocation decisions can lead to overreliance, loss in competencies or motivation deficits (see e.g., Bainbridge, 1983; Onnasch et al., 2014).

To anchor a human-centered perspective in HRI design, task allocation can be the decisive regulating screw. To fulfill a broader task, it is necessary to perform certain subtasks, for example fetching parts together, assembling and controlling the quality afterwards. Task allocation is the critical, often pre-planning process to determine who is responsible for which subtasks. As it is such an important process and an enabler of HRI, it needs systematic examination, as well as rethinking. Reviewing the literature on allocation, Challenger et al. (2013) point out that a macroergonomic, strategic approach toward designing allocation processes is missing and overlooked due to a microergonomic focus on tasks. Beyond their definition of task allocation as an explicit process in systems design and development and their systemic view on it (Challenger et al., 2013), there is not much research looking at this process in its entirety or at how the allocation process affects people in the work system. This neglect can lead to inefficient allocation decisions and HRI systems failing because basic psychological principles and human needs remain unconsidered.

We therefore focus on the process leading to an allocation decision in HRI and describe process characteristics as well as their psychological implications from a perspective of self-organizing socio-technical systems (see e.g., Naikar, 2018). This means looking at allocation as an *ad hoc* process that allows for inclusion of the workers in the allocation decision. In this article, we develop a model that structures this *ad hoc* task allocation process and connects it to psychological outcomes related to motivation and task regulation. The process model helps in the consideration of factors that are important for designing accepted and humane systems in which humans and robots can work together efficiently.

## *Ad Hoc* Task Allocation in Human–Robot Interaction

A vital step toward describing allocation processes in HRI is to delineate the subject area of the model. We refer to it as a process model of *task allocation*, describing “the distribution of tasks between humans and machines within the work organization” (Older et al., 1997, p. 151). In the allocation context, also the term *function allocation* is used, describing a “decision-making process and method that is used during the design life cycle of complex systems to distribute the system functions […] among all agents in a team, namely humans and automated systems” (Joe et al., 2015, p. 1226). According to, e.g., Older et al. (1997), the terms are often used interchangeably. In their newer definition, the authors even speak about the “allocation of functions and tasks” as the same thing (Clegg et al., 2000, p. 238). We decided to use the term task allocation for our model to underline the relatedness of the distribution to a specific task with a certain objective, but will include literature on function allocation just as well because of the closeness of the constructs. What is considered in this study is, as in the definitions, only the allocation of subtasks to the different agents in the system, i.e., the workers and robots. To fully plan a production process, other factors such as interdependencies between subtasks or their order need to be considered, but those are not in the focus of this article.

**FIGURE 1:**
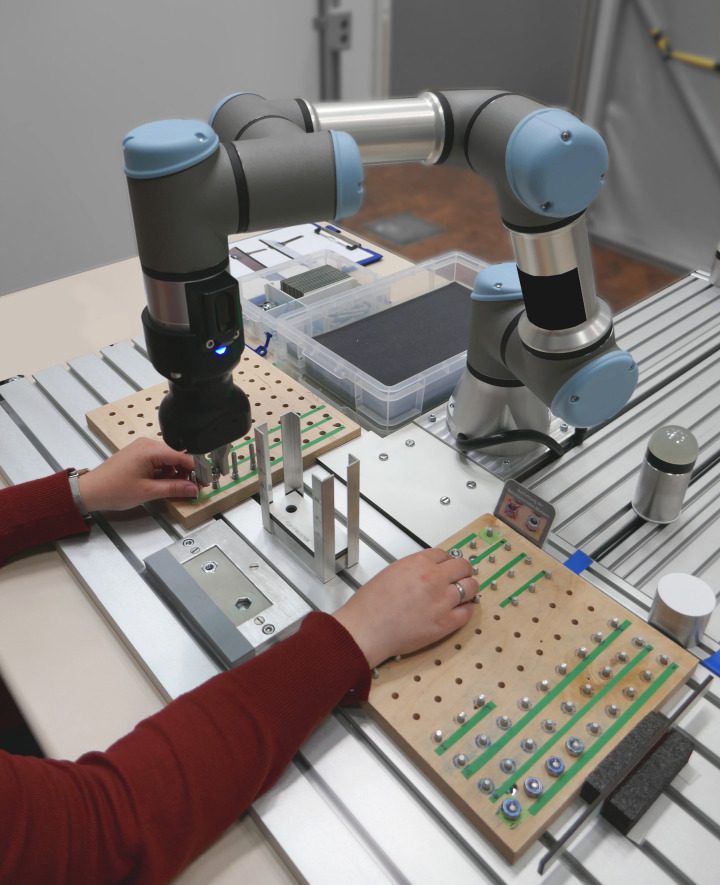
A collaborative robot in an HRI setup.

Task allocation is described by Abdallah and Lesser (2005) as a problem appearing whenever a task cannot or is not supposed to be conducted by one agent alone. The field of task allocation has been dominated by the question who, man or machine, can perform a certain operation better (e.g., MABA–MABA principle by Fitts, 1951; Dekker and Woods, 2002) and the aspiration to automate everything possible and leave the “rests” for humans (McCarthy et al., 2000). This thinking is still quite present today, but for current developments in the production sector toward faster, more individualized production in more flexible settings able to be rearranged they are probably not appropriate. The upcoming *collaborative robots* (see e.g., Figure 1) can be a technological support for mastering these new affordances.

These collaborative robots are easily programmable, lightweight and small and can work together with humans in a small space and without physical barriers. This technology offers new and flexible ways of integrating robots into production processes and creating cooperation and collaboration as relevant forms of work (for further reading see Schmidtler et al., 2015). Cooperation, which is relevant in our case, is described as human and robot sharing one workspace and working there at the same time on a shared goal. This form of interaction implies a closer integration of the human in using the robot and potentially intertwined work orders and/or task execution. To raise acceptance, decrease fear and prejudices, to support a realistic assessment of robotic capabilities and empower people to learn from interacting with collaborative robots, new forms of task allocation processes are needed to realize successful cooperation.

### Self-Organization in HRI

A new approach toward task allocation in HRI can be that of a self-organizing sociotechnical system in which tasks are allocated *ad hoc* instead of pre-planned. Self-organization is defined as “a dynamical and adaptive process where systems acquire and maintain structure themselves, without external control” (de Wolf and Holvoet, 2005, p. 7). Originally, self-organization in the field of robotics refers to one or multiple robot(s) behave in a certain way due to their interaction, e.g., one robot automatically collecting goods in reaction to another robot in the system building parts from these goods. The robots adapt their behavior to that of the other system elements.

The concept of *self-organizing sociotechnical systems* goes a step further in the understanding of self-organization, including both humans and machines. Together, they form a sociotechnical system that regulates itself. The concept is based on the observation that new organizational structures evolve in reaction to environmental changes, when sociotechnical systems are not pre-planned and coordinated centrally (Naikar, 2018). Fuchs describes the human as the central moment in self-organizing social systems: Their participation and cooperation can enhance knowledge usage and synergies (Fuchs, 2004). His concept has been further developed and transferred to the field of human–automation interaction and that of socio-technical systems. Naikar (2018) argues that the limits of the “who can do what” can be overcome by allowing the system’s actors degrees of freedom for behavior and by creating integrated system designs (Naikar and Elix, 2016). Although this concept originates from the coordination of complex cognitive work, we transfer it to the case of modern production settings with (a) increasingly complex demands, (b) dynamically changing production setups, and (c) the need to keep the workers in the loop, to preserve their qualification and to motivate them to work closely together with robots (possibly instead of colleagues). We therefore use a work psychological concept of self-organization in the sense of a system of human(s) and robot(s) coordinating without external control, adapting to each other’s behavior and the system’s state and therefore cooperating not only in task execution but also in task allocation.

Closely related to that idea of self-organization and integrated system design is that of *ad hoc* task allocation. Whereas task allocation is traditionally understood as a pre-planning process – as to Hacker the task that is prior to all other task segments (Hacker and Sachse, 2014) – we want to go beyond common practices and approach more flexible concepts like that of dynamic task allocation. As to the review of Older et al. (1997), there are numerous forms of dynamic allocation ranging from those automatically adapting to situation (see e.g., Greenstein and Revesman, 1986; Scallen and Hancock, 2001) to those being adaptable by humans (see e.g., Kidwell et al., 2012). In this context, *ad hoc allocation*, sometimes referred to as real-time allocation, describes, in our understanding, those task allocation decisions that are (a) integrated into the working process – i.e., not a timely and spatially separated decision, (b) done by the workers involved in the working process, and (c) open toward changes throughout the working process, i.e., adaptable and/or adaptive. They are to be understood as behavioral processes with feedback loops that entail changeability throughout the working process.

### Concept Behind the Allocation Process Model

Based on these general concepts, we developed a research model to describe and examine *ad hoc* task allocation in HRI. This model is an important first step toward systemizing research on task allocation in HRI from a work psychological point of view. It is supposed to (a) give a structure for describing an allocation process that is based on established theory and research, (b) show relevant psychological effects of allocation processes and decisions that need to be examined in an HRI context, and (c) visualize critical spots for system designers to either adapt or newly develop *ad hoc* allocation processes that consider psychological aspects.

The model is first and foremost a means for researchers, as there is a substantial lack in the consideration of allocation processes on a meta level on the one hand (Challenger et al., 2013) and in the consideration of cognition, affect and behavior of the workers in a cooperative setting on the other hand. It is not developed as a method for finding the optimal allocation solution and so not primarily suitable for a direct application in practice. Nevertheless, it can serve as an inspiration for system designers implementing robots into work systems to more widely consider a human-centered approach in HRI and reflect on possible psychological effects a certain allocation practice has on the workers and, in the long run, on the functioning of the interaction.

### Methodological Approach Toward the Model

Theory building can be understood as finding a scientifically grounded general principle to explain a certain phenomenon (Merriam-Webster, 2020). Korte (2016) advocates for a pragmatic approach including multiple methodologies, especially in applied disciplines. To develop our research model, we therefore approached the existing body of literature on task and function allocation, HRI and decision-making from different disciplines like engineering, psychology, or management. We then selected relevant literature to develop a procedural model of *ad hoc* task allocation and to deduce outcomes related to psychological wellbeing relevant to task allocation processes in HRI. The model shall work as a guideline for future research.

Our literature research and selection was done subsequently in multiple periods, mainly between May and June 2018, with multiple additional phases in April and May 2019 and February and April 2020 to keep literature up to date. We chose to not compile a systematic review, as there is not much specific empirical research on the psychological effects of (*ad hoc*) task allocation in HRI yet. We instead started with literature on allocation in human–robot interaction and then broadened the scope as we identified a lack in both psychological and process-related perspectives in this research area. EBSCOhost and Google Scholar were used to look for relevant papers and proceedings under key words such as task and function allocation, scheduling, human–robot interaction, cooperation and collaboration, human–automation interaction and decision support systems. Research on psychological constructs was based on keywords like (work) satisfaction, decision perception, work design, psychological outcomes as well as on specific constructs like mental effort that we assume to be influenced by task allocation. Afterward, we also used sources found in the identified literature to broaden our literature base. We ended up considering literature from the fields of (system) engineering, theoretical and empirical psychology, robotics, computer science, automation, management, and medicine.

To develop the process part of the model, we then had to integrate our insights from the research. Starting with basic theories of human behavior and task execution (task regulation theory and Rubicon-model, see section “An Example of an *Ad Hoc* Allocation Process”), we described the generic steps of an *ad hoc* task allocation process in HRI. We then integrated findings from our research on task allocation to refine different aspects of the allocation process and integrated those into the more general steps. The process part of the model is depicted in Section “An Example of an *Ad Hoc* Allocation Process.”

Task regulation is defined as a mental process to regulate a person’s task-related behavior by goal-setting and feedback: A central idea of the task regulation theory is that psychological reactions are a mean to regulate behavior and therefore determine what a person is doing (Hacker and Sachse, 2014). This idea is vital for the second part of our model: the psychological consequences of task allocation processes. We expect the single process steps of task allocation to lead to specific cognitive and affective reactions that each influence human behavior to the extent of willingness to cooperate with a robot, amount of effort invested into the work, learning behavior or even sabotage of the robot. This shows the importance of looking at the psychological effects of allocation design and allocation outcomes.

For each step of the allocation process, we looked for possible psychological outcomes on the perception of the self, the task and the interaction that have not yet been broadly considered in research on task allocation in HRI. We studied the literature on corresponding steps in decision-making in general or in work design and looked for constructs like self-efficacy or process control with possible relevance also for allocation processes. These psychological constructs were then added as consequences of the allocation process steps. Wherever possible, we included connections between these constructs from studies and connected them with arrows to illustrate the mutual dependencies between them. The psychological consequences are depicted in detail in Section “Psychological Consequences of Allocation Processes.”

As a whole, this model is to be understood as a hypothetical approach toward psychological effects of task allocation in HRI. As theories, models or even studies on this specific topic are still quite rare, we hope that our integration of literature on related areas and on task allocation from non-psychological points of view helps to examine the process and its effects systematically.

### An Example of an *ad hoc* Allocation Process

To clarify our idea of an *ad hoc* task allocation in human–robot interaction, we want to give an example of a potential allocation process: the initial situation is that of a production worker responsible for making switchgears together with a collaborative robot in his workspace. Both can do certain tasks in the production process, with some of them only being executable by the robot (e.g., because parts that need to be lifted are too heavy for the human), some only executable by the human worker (e.g., fixing small signs to non-rigid cables) and some both could potentially resolve (e.g., sticking bridges into functional parts to connect them). Especially the latter are interesting, as they can be allocated dynamically to human and robot throughout the working day.

The worker chooses his first task and starts the execution. Because the workpieces, workstations and the robot are interconnected in a cyber-physical system (=CPS), the robot knows what the person is working on and starts with the next task needed in parallel. If the worker needs additional material to continue during his tasks, he can assign the robot to collect it from the warehouse. After returning, the robot expects an allocation input from the worker or starts with a new task automatically after some time. When problems occur or the quality of the robotic execution is not sufficient in the eyes of the worker, he can take over the task from the robot and assign another task to it. The worker can as well allocate tasks to the robot that he has executed himself, e.g., due to a high strain of the hands after some time. In this scenario, the worker and the robot are in constant communication about the tasks that need to be resolved and their allocation.

The iterative process allows for adaptations at all times and a fluent process of producing and redesigning the allocation. At the end of the working day, the worker and the robot jointly completed their task of building marshaling panels – and they self-organized dynamically to be able to complete the task.

We assume that this kind of scenario does not only meet modern-day expectations on a flexible, partially automated production, but also is psychologically valuable as it contributes to a humane work design that keeps or even leverages the workers’ qualification levels, opens freedom for job crafting and pushes a positive self-experience as an important part of a human–robot team.

## The *Ad Hoc* Allocation Process Model

The model we developed and illustrated in the example first describes the process of an *ad hoc* allocation between worker(s) and robot(s). As a base for the model, we used the task regulation theory by Hacker and Sachse (2014) that structures work processes into an objective order that is redefined into a task with specific goals by the person having to execute it. The task is then executed through a number of actions and operations which then lead to the fulfillment of the initial order. We follow this basic principle for describing the task allocation process.

**FIGURE 2:**
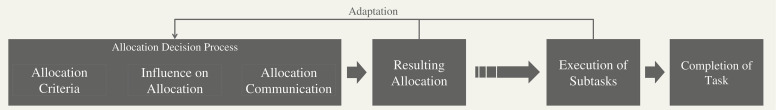
The process of *ad hoc* task allocation in human–robot interaction.

Abdallah and Lesser’s (2005) general description of allocation problems as a division of a task into subtasks, which are then allocated to the agents at hand in a decision process, inspired us to look into decision making for the process model. That is why we also integrated the Rubicon-model (e.g., Heckhausen and Heckhausen, 2018), an influential model for explaining volition in the decision-making context, structuring processes of motivated action: After a deliberation phase, comparable to taking an order and redefining it as an own task, the planning phase starts, ending with the intention initiation. This then leads to volitional actions, until certain outcomes are achieved that are evaluated (Heckhausen and Heckhausen, 2018). These theories lead to the essential four steps of the process model depicted in Figure 2.

### Allocation Decision Process

The allocation decision process is shaped by three essential characteristics: the criteria used for allocation, the influence the worker has over the allocation and the way the allocation is communicated. In our cooperative production example, decision criteria are manifold, but the realization of a workflow without waiting times (for this, the robot adapts by automatically choosing a task when it is not allocated one by the worker) and an optimization of strain for the worker are central. The worker as well as the robot have influence on the task allocation, with no external agent involved and task allocation by the robot is communicated via an audio output informing the human shortly on which task the robot is about to execute. A tablet connected to the robot shows the current production status and a rating on the recommendations for each task available. These three components shape the allocation decision process on their own as well as in interaction with each other and are described in the upcoming sections.

#### Allocation Criteria

The criteria used in an allocation decision shape the allocation result to a great extent. Which criteria are applied depends on who is involved in the allocation (see further below) and the goals of the work process. Especially in a variety of publications on optimization of task allocation in HRI, but also on more general planning algorithms, objective goal criteria are presented upon which algorithms base their decisions. The field of project scheduling problems (PSP) deals with planning under restricted resources and a given time frame (Chen et al., 2014). In production, the problem of minimizing the costs for resources for a fixed date, a resource investment PSP, is to be considered. When there is more than one goal to be reached in the planning process, multiobjective optimization (MOOP) approaches can be used. Transferred to the context of hybrid production, this means that not only the reduction of work costs and production time is considered, but also aspects like computational effort (Gerkey and Mataric, 2016), competence retention, appropriated workload or change in tasks (Chen et al., 2014).

Approaches mindful of human needs are rare, especially in the practical application (Older et al., 1997). With Wilcox et al., 2013, algorithmic planning can adapt robotic actions as to human preferences and changing time frames [reported by Pinto et al. (2017)]. Castro et al. (2017) consider uncertainty for example through hesitation or individual responses to robotic actions in automated planning.

#### Influence on the Allocation Decision

Historically, task allocation “involves the design team considering each task and the relative advantages and disadvantages associated with that task being performed by the man, or by the machine” (Stanton et al., 2005, p. 485). As we in our model consider task allocation as an *ad hoc* process involving the worker, we use a continuum between the worker and an decision support system (connected to the robot) and describe it as influence over the allocation (decision) throughout this paper. The shared decision can be located on any point on the continuum between a worker deciding on their own and a fully automated decision and reflects the idea of a self-organizing sociotechnical system (Naikar, 2018). This concept also acknowledges the practical limitations of worker involvement by integrating a system in the allocation decision that sets the (often economical) borders of an allocation decision, reflecting the demands of the management.

Parasuraman et al. (2000) differentiate ten *levels of automation* for decision and action selection. They range from a solely human decision (level 1) to a support of human decision via automation (levels 2–4), an automated decision with possibilities for influence or information (levels 5–9) to a solely automated decision (level 10). This concept could be used to describe the degree of worker and system influence as it is very well established. Nevertheless, its focus on the automation-side makes it not fully suitable to design decision processes from a human-centered point of view. The specific number and differentiation of levels, as Wickens (2018) himself states, is not mandatory and can be defined differently for different functions. An alternative approach is that of Cummings and Bruni (2010): they differentiate collaboration, specifically in planning resource allocation, into five levels. A level of 2 is a sheer human decision, whereas at level 1 the human is supported by automation, but still has the greater part in the decision. Level 0 describes an equally shared decision, −1 one that is shaped more by automation und −2 one that is made only by automation (Cummings and Bruni, 2010). These two general automation level models can also be used to describe human–machine cooperation in task allocation and the amount of mutual influence.

Studies on automation-supported allocation have shown that a merely automation-generated allocation solution can lead to a loss of situation awareness and automation-biases (e.g., Parasuraman et al., 2000). Hence, Cummings and Bruni (2010) state that a collaboration of human and automated systems can improve the performance of both the operator and the decision system, especially under time pressure and uncertainty.

Studies investigating workers’ perception of allocation processes in a collaborative context are quite rare. Munzer et al. (2017) find that a self-learning, autonomous and initiatively task-executing robot is perceived as more helpful and is preferred in collaboration. Castro et al. (2017) compare a human-led and a robot-led experimental setting in which humans and robots have to execute a task based on the division of labor. Gombolay et al. (2015) show that satisfaction of the human can increase when a semi- or fully autonomous system takes responsibility for planning. The question evolving is whether people are actually more pleased with the allocation process itself compared to making their own decision. Or is it just the higher productivity (Gombolay et al., 2015) and fluency of processes (Munzer et al., 2017) resulting from automation-generated planning that makes them more satisfied? An indication for this could be the findings of Cummings and Bruni (2010): They show how people even try to participate in a resource allocation task that can be highly automated, choosing a manual adaptation of automated decisions or comparing an automated to a manual solution instead of relying on the system. This behavioral pattern of influencing automated decisions can be a hint at the preference for influence on allocation decisions.

#### Allocation Communication

The third decisive characteristic of the allocation decision process is the communication of the task allocation or of the support given in the allocation process leading to the decision. A lot of research on communication of decisions comes from medicine, examining the interaction of patient and doctor: The concept of shared decision making in healthcare differentiates between more or less risky and uncertain situations, with each needing a different degree of either information or even sharing of decision-making (Whitney et al., 2004). Politi et al. (2011) find that the expression of uncertainty of a decision by a doctor leads to less satisfaction with the decision. It is not certain if this finding is applicable also to the field of communicating allocation decisions by a robot or an automated system. Though one would expect high expertise in both contexts by the communicating agent, doctoral decisions are often such one’s life depends on and therefore uncertainty elicits fear. In the case of task allocation, admitting uncertainty might even elicit more satisfaction as the counterpart “knows what he gets.” Research on decision communication in business and its consequences is, however, quite rare (Mykkänen and Tampere, 2014).

Additionally, there is the field of software interaction with users, which is partially transferrable to the communication of decision (support) by an assistance system. Johannsen et al. (1983) ask some questions decisive for the design of successful human–machine interaction: which information is needed, how is it collected, structured and analyzed before being displayed, how should judgments be formulated and how should the transfer of information be realized in decentralized situations? For communication of decisions, especially the last three questions are relevant. Carneiro et al. (2020) e.g., point out how important communication is for group-decision making in their development of a group decision support system – which resembles decision making in cooperation with a system in the involvement of multiple agents and the need to exchange information. Seong and Bisantz (2008) show that providing operators with meta-information, the performance of their cooperation with a decision aid improves as well as their trust in the system.

### Resulting Allocation

The final allocation of subtasks to workers and robots is the result of the allocation process. This result is an allotment of the previously defined subtasks on humans and robots and forms the basis for the upcoming task execution. In the example, the initial result of the allocation is the worker assembling electrical components while the robot lifts a metal base and the walls for the switch cabinet to the worktop. The further steps of building the switch cabinet are allocated later in the production process.

Gombolay et al. (2015) show the resulting task allocation between humans and robots in their experiment: Participants allotted more tasks to themselves compared to when a team partner or the robot made the allocation decision. This effect is in line with the phenomenon of planning fallacy (Kahnemann and Tversky, 1979), stating that the time one needs for a task is systematically underrated, whilst the time that others need is overrated. This shows that the allocation solution is highly dependent on the agent of allocation and the criteria they apply.

From an economical point of view, the result of an allocation can be judged by the efficient use of resources. Appropriate utilization of a robot is important for quick amortization of investments. At the same time, the human also needs to be appropriately deployed to his/her capacity, with short waiting times but also just as many tasks as he/she can handle. This also holds from a psychological perspective.

### Execution of the Task

When an allocation decision has been made, the agents can execute the single subtasks. In our switchgear production, human and robot execute their tasks in parallel, with the worker combining different work pieces to make terminal blocks and the robot driving to the stockyard, picking up the metal pieces, carrying them to the joint workplace and lifting them to the worktop.

Insights into task execution in the context of allocation are given in a study by Castro et al. (2017): in trials where the human was responsible for the allocation, very different waiting times resulted for the robot, which were then adjusted over the number of trials. The trials allocated by the robot were significantly faster – if the robot sets the pace, team efficiency is higher (Castro et al., 2017).

### Adaptation of the Allocation

Our *ad hoc* allocation model also includes feedback loops in the form of possible adaptations of the allocation throughout the process to acknowledge the need for flexibility of production processes as well as the workers’ needs of adaptations to their current situation. This adaptation can be performed in different ways. The worker has, e.g., built terminal blocks for about an hour now and feels increasingly tired in pressing the terminals to the top-hat rails. During execution, he therefore decides to reallocate the terminal block-building to the robot and to take over another task instead, which he does by using the robot’s tablet to advise the new task to it. Continuing their work, the robot continuously logs the production output and after some time registers that there are now enough terminal blocks for the switchgear to be built. It then analyses the current production situation and defines crimping cables as the next most important task in keeping the workflow. The robot moves to the crimping station and starts the execution there, informing the worker with a short audio note.

Smith et al. (2020) develop a method to adapt task allocation according to current worker capabilities by monitoring throughout task execution. Such approaches represent an *adaptive* process with feedback loops based on pre-set objective criteria that, at a certain point in time, automatically re-allocate tasks accordingly. Another approach is to make task allocation *adaptable*, i.e., changeable for a person in a non-automated manner. In the context of automation, Kidwell et al. (2012) examine the influence of adaptivity and adaptability, coming to the conclusion that adaptability increases workload, but also performance criteria and operator confidence. Müller et al. (2017) state that robot operators have to adapt their workload according to their current capabilities and develop a system open to changes in task allocation by the operator on the shopfloor.

### Completion of the Full Task

The completion of the full task is mostly about the degree to which different performance criteria are achieved by the execution of the combination of subtasks. The desired value that the result is compared to can be determined from different perspectives, for example from an economic, process-oriented or human-centered point of view. From a psychological perspective, it is especially interesting to see how the employees experience the fulfillment of tasks and which criteria they use for their evaluation. In the example, the full task is completed when the clients order of the specific switchgear is fulfilled. For the worker, this has been successful when the chosen task allocation led to a high-quality product, with the testing unit not finding any malfunctions and he and the robot having worked fluidly together in building a product in time.

In research on strategic business decisions, it has been pointed out that different processes lead to different decisions and this in turn leads to different outcomes, so not all decisions are of the same fineness (Dean Jr., and Sharfman, 1996). This quality of a decision depends on external influences, but is also influenced by the processes leading to a decision (Dean Jr., and Sharfman, 1996).

An experiment by Cummings and Bruni (2010) about automation-supported resource allocation shows that the performance of the allocation differs depending on the degree of collaboration in decision-making. A scenario with only little participation of the human resulted on average in a solution that was a little less good than in the scenarios with equal participation of human and automation or with a higher human share (Cummings and Bruni, 2010). This shows not only the influence the process characteristics have on one another, but also the opportunities a self-organized allocation process involving the worker has to improve work.

## Psychological Consequences of Allocation Processes

**FIGURE 3:**
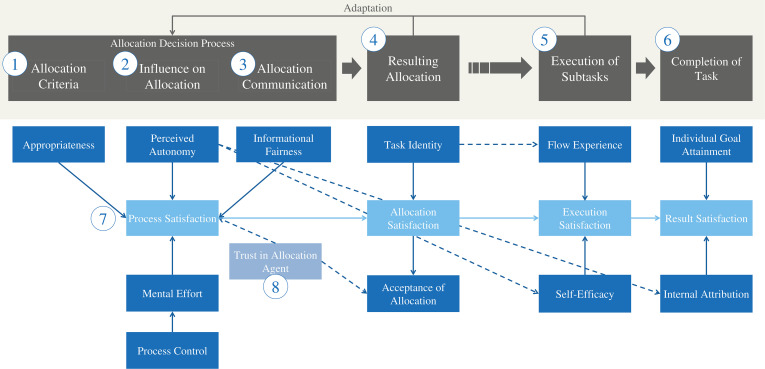
Psychological effects of the *ad hoc* task allocation process.

After having structured the allocation process into distinct steps, we now consider psychological constructs important for perceiving, experiencing and assessing task allocation (see Figure 3). Those constructs identified in our literature search (see section “Methodological Approach Toward the Model”) are potentially important psychological consequences of the design of the allocation process. They are assigned to each of the elements of the allocation process model (see Figure 3) and are presented in a sequence following the numbers in the figure. We drew arrows between constructs that we found to be connected to one another. The straight lines visualize connections of outcomes to the satisfaction facets, whereas the dotted lines refer to connections between different outcome variables. Those will be presented as propositions in Section “Propositions – Linking the Psychological Consequences.” The links we drew might not be exhaustive, but sketch the most influential correspondences in our eyes in regarding allocation consequences.

As introduced above, all constructs are crucial for psychological regulation and decisive for a functioning cooperation between humans and robots that needs trust and acceptance of the robot and the will to cooperate to work out properly more than any other human–machine interaction (e.g., Hancock et al., 2011). In the following, all the constructs identified as relevant to the experience and evaluation of the *ad hoc* allocation process are presented in further detail and are illustrated by connecting them to the example.

### Appropriateness of Criteria

When it comes to the decision criteria, perceived appropriateness is decisive. A judgment on *appropriateness* stems, following task regulation theory (Hacker and Sachse, 2014), from the accordance of one’s own goals for task execution with the criteria used for judging the execution. If the criteria are perceived as appropriate and match with one’s own goals for this action, a decision or recommendation by someone else probably has a greater chance of being accepted and of being followed through. Referent Cognitions Theory (Folger, 1986) illustrates this: in a situation where outcomes are allocated by a decision maker, anger is greatest when the person affected could have expected better outcomes from another, more appropriate decision method that the decision maker could have applied (Cropanzano and Folger, 1989).

In our example, the worker would rate appropriateness low if the robot allocated tasks not based on criteria like efficiency or competencies but, e.g., on how much pressure it can put on the worker to work faster and faster. The worker could even be in anger as he could have delivered better-quality work (which, as we know, is one of his personal goals for work) not being pressured by the robot.

### Autonomy, Process Control, and Mental Effort

Task allocation processes that involve workers up to the extent of a self-organizing sociotechnical system aim at giving the workers back some of the control and freedom lost in sharing the own tasks with a robot. Hence, the design of allocation processes, and especially the configuration of influence over the allocation decision should influence the perception of autonomy and process control, as well as the worker’s mental effort needed for the allocation.

*Autonomy* is one of the most intensely researched constructs in work psychology and is unanimously considered a decisive resource in work life as well as an important human need in general influencing well-being according to self-determination theory (Ryan and Deci, 2000). Models like the job characteristics model (Hackman and Oldham, 1976) or the expanded work design model (Humphrey et al., 2007) contain autonomy as a core variable. Hackman and Oldham (1976) define it as the grade to which work offers freedom for the person working in planning, setting processes and executing. Humphrey et al. (2007) differentiate autonomy into the three facets of planning, method and decision autonomy. It has correlations to intrinsic motivation, work effectiveness, satisfaction with one’s own development und absenteeism (Humphrey et al., 2007).

How much autonomy can be experienced throughout the allocation itself is mainly determined by the distribution of influence on the decision process. Especially decision and planning autonomy are influenced by the control one can exercise on the allocation of tasks (see further below). The feeling of autonomy should then have an influence on self-efficacy (e.g., Wang and Netemeyer, 2002) and the attribution of success or failure to task execution.

In their research on procedural justice, Thibaut and Walker (1975) differentiate between process and decision control: decision control, the objectively given responsibility for the decision, leads to decision processes being more fair, even if the decision is not binding (Lind et al., 1983). If someone or something else is in charge, process control becomes more important. *Process control* or voice is the ability of the affected person to express his/her opinion about the situation and the decision (Leventhal, 1980). Decisions not to the advantage of the self are experienced fairer if a procedure with voice led to that decision (Bies and Shapiro, 1988). A study from 2018 examines the influence of decision agent and procedural justice in human–machine interaction: Ötting and Maier (2018) find main effects of procedural justice on work satisfaction, organizational commitment, organizational citizenship behavior, cooperation and contra-productive behavior.

A higher degree of process control should go along with higher demands resulting from the execution of this control – i.e., a higher *mental effort*. Demands of work are physical, organizational and social characteristics that need physical or mental power (Demerouti et al., 2001). From them, a psychological strain evolves, which is dependent on the habitual and current attributes and strategies for coping (DIN EN ISO 10075-1, Din Deutsches Institut für Normung,, 2018). Wieland and Hammes (2014) distinguish a positive from a dysfunctional facet of strain: Whilst the first, eustress, leads to self-efficacy, positive emotions, and flow experiences (see e.g., Csíkszentmihályi, 2012; Wieland and Hammes, 2014), dysfunctional strain, distress, can lead to negative psychological outcomes.

The complexity of task allocation, which demands cognitive operations of the person deciding, depends on the situation, resources at hand, knowledge of the system and type and amount of information given. Assistance systems, so-called decision-support systems, can reduce cognitive load (see e.g., Glasspool et al., 2007). Onnasch et al. (2014) report studies on automation level and subjective workload: Six out of the twelve studies evaluated show strong negative effects, contributing to the overall effect and indicating that a higher automation level might reduce mental workload. But assistance systems can also increase strain if they are complex in themselves and e.g., offer more information than the actual environment.

The worker in our example experiences a high degree of decision autonomy as he can allocate the tasks as he wants, being able to adapt the allocation at any point. This also gives him high process control, even in the case of the robot adaptively taking over tasks by itself, as he can always see the reasoning basis for the robot’s decision on the tablet and can step in and change the allocation. To allocate the tasks, he needs to invest some mental effort to make a sensible allocation decision. But as he is supported by the robot self-allocating when no input is present, this automation support can reduce his mental effort and help balance the strain put on him.

### Perceived Informational Fairness

When a worker allocates tasks in HRI under support of a system, there will always be some form of communication of the system with them. The importance of adequate communication for motivation and behavior is shown in an experiment by Langer et al. (1978): they asked people to let somebody go first on a copy machine and only gave minimal reason for this. People complied more often when there was a reason, partially even if information was placebic. This is probably because these information evoke the perception of fairness. *Informational fairness* describes the open handling of information. It is experienced when the affected persons have access to all the information relevant in a decision (Wirtz, 2014), or, as in the example, heuristically judge to have the information needed. This perception of fairness is partially elicited by system or robot transparency in the case of human–robot interaction, with the interaction partner delivering the information necessary to feel fairly treated information-wise. Bies and Moag (1986) and Greenberg (1993) frame two rules of informational justice: honesty, which is the honest description of processes, and justification, which is the accurate explanation of processes. In a series of studies from Binns et al. (2018), many participants perceived an algorithmic decision as odd, impersonal or even dishonorable, whilst for others justice was not a topic, as the system was perceived to only do what it was supposed to do. Quantitatively, no differences in the experience of justice could be shown between different explanation methods for this decision.

Referring to the example, the worker perceive the allocation process as fair because the process of task allocation is described clearly and works, whenever the robot behaves autonomously, as to predefined criteria that are transparent to the worker. The user interface on the tablet presents to him all information relevant to experience the process as fair.

### Task Identity and Acceptance of the Allocation Result

The allocation of some tasks to the human and some to a robot (i.e., the resulting allocation) has an influence on the task completeness the human can experience. Hacker and Sachse (2014) use the term “completeness” for tasks: sequentially complete tasks contain all steps from preparation through to organization and execution control of action results. Hierarchically complete tasks demand psychological regulation on all levels, that is sensomotoric as well as knowledge-based and intellectual actions (Adams, 1965). The construct of *task identity*, as used for example in the Work Design Questionnaire (referring to the Job Characteristics Model) (Morgeson and Humphrey, 2006), is an equivalent to completeness, defined as the extent to which a job is a whole piece of work with distinct results (Sims et al., 1976).

Any allocation of subtasks to more than one agent will inevitably narrow down sequential completeness to a certain degree. Nevertheless, a task can be complete when a product is accompanied through the production process from start to finish, even if not all operations are executed by the same person. If a person has knowledge about the whole process and influence on the subtask he or she executes in a certain situation, a task can still be perceived as complete, even if sequential completeness is not fully given.

Depending on the result of an allocation, employees might be willing to accept the decision, rate it as appropriate and put up with it to a greater or lesser extent. *Acceptance* of the decision leads to readiness to behave according to the allocation and to execute the allocated tasks. Price et al. (1964) examine acceptance of automated landing systems by pilots: they find that people accept the role they were assigned to when they can execute the abilities they consider important, when the role leaves methodological freedom and when it allows learning (Price et al., 1964). The acceptance of long-run steering tasks is better than that of decision functions by an automated system (Price et al., 1964). As van Swol and Sniezek (2005) find, the acceptance of expert advice is dependent on the trust in this expert (for a discussion on trust, see section “Satisfaction With the Steps of the Allocation Process”). It shows that not only the content characteristics of an allocation solution are relevant for acceptance, but also context factors like task characteristics or expertise involved.

The task outlined in the example is not sequentially complete from an objective point of view, as the worker does not execute all steps of it, but high task identity can nevertheless be perceived, as building a switchgear is a whole piece of work and the worker is involved in most steps to solve this tasks, be it by executing them itself or by allocating it to the robot. Therefore, he knows and understands the whole production process, which is a resource to him. His acceptance of the allocation whenever the robot allocates a task itself is high, as he does not believe that the robot is to be distrusted and is agreeable with the allocation decision most of the time.

### Flow Experience and Self-Efficacy

An aspect vital for motivation within the execution of tasks is *flow experience*. It is a state of optimal demand in which one is totally absorbed by a task (Csíkszentmihályi, 1990). The experience of flow is intrinsically motivating and leads to positive affect and the readiness for staying with a task (The European Flow Researchers Network [efrn], n.d.). Nevertheless, it is bound to some prerequisites that are influenced by task allocation: it needs a challenging task that can be mastered with the abilities of the person (Csíkszentmihályi, 2012). An allocation process can be suitable to a greater or lesser extent for creating such a match. Leeway in choosing the tasks can facilitate the choice of matching tasks: Kowal and Fortier (1999) find such correlations between autonomy and flow experience with athletes. There are also hints for the role of task identity: according to Csíkszentmihályi (1997), important, clear goals are vital for flow experience, as well as knowing exactly what is to be done. In a task that is complete and not fragmentized, which is vitally coined by the allocation result, this happens more easily.

Closely connected to the experience of flow (see e.g., Salanova et al., 2014) is *self-efficacy*. It describes the beliefs about one’s own abilities for reaching a designated performance (Bandura, 1994). If someone feels self-efficacious with the execution of their work, they also try to handle more challenging situations, get absorbed in a task more easily and can therefore reach their own goals (Bandura, 1994). That is why self-efficacy is a decisive belief shaping the execution of tasks. For experiencing self-efficacy, not only personal prerequisites are important, but also the design of tasks. Kowal and Fortier (1999) and Wang and Netemeyer (2002) could prove positive correlations of experienced self-efficacy and autonomy. Parker (1998) shows that measures for job enrichment can increase self-efficacy in a broader sense. So we can assume that also the design of the allocation process with scope of influence for the worker as well as different resulting allocation solutions can influence experienced self-efficacy. A study by Kidwell et al. (2012) shows that the application of an automated system that is adaptable in its degree of automation, i.e., in which tasks it takes, leads to increased confidence in task execution by the participants. These findings support the hypothesis.

As the worker in our example enjoys high autonomy in allocating the tasks, he can chose those tasks that allow him to experience flow, to forget about time and just be focused on the execution. What helps with this is task identity because knowing the whole task creates clear goals to work toward. He can also gain self-efficacy from the work process, as mastering challenging demands like allocating the tasks efficiently contributes to producing switchgears successfully. The worker becomes more and more self-efficacious in the working process and in using the robot, which strengthens his personal resources and makes him confident in mastering his work.

### Individual Goal-Attainment and Internal Attribution

One of the subjective criteria for evaluating achievements in work is task fulfillment. This is, according to the redefinition paradigm (Hackman, 1969), the inner model derived from the factory order containing the realization conditions and a depiction of the goal (Hacker and Sachse, 2014). In the regulating process of orienting behavior, goals are formed in accordance with one’s own demands, values and performance evaluations (Hacker and Sachse, 2014). The attainment of these goals is supervised throughout and after the execution of the task and serves as a basis for further regulation. To this extent, the *attainment of self-set goals* is a vital source not only for personal satisfaction and experiences of success, but also for the motivation of further work behavior.

The goal-attainment can be attributed either internally or externally. The origin of this construct lies in the explanation for the behavior of others: the belief that behavior is shown out of the person itself, for example because of their personality, and not resulting from the situation, is called *internal attribution* (Heider, 1977). In relation to one’s own performance, Weiner further developed this construct and describes the causes for failure and success on the three dimensions location, stability and controllability (Weiner, 1986). An internal location describes the allocation of performance responsibility to oneself and is strongly related to controllability of the situation in its consequences. As to Weiner’s theory of emotion emergence, after the assessment of causes of an issue, an attribution of responsibility only arises when the cause is seen as controllable (Weiner, 1986). It means that an internal attribution of a work result leads to experienced responsibility for the result only if it has been controllable through one’s own actions. The availability of autonomy makes one’s own actions more controllable and allows for internal attribution of work results (Hacker and Sachse, 2014). As well, this internal locus of control is closely related to flow experience (Keller and Blomann, 2008) and self-efficacy, whereas the latter is more strongly connected to behavioral intentions in the future than to actual behavior as is perceived controllability (Terry and O’Leary, 1995).

The worker from the example redefined the factory order of producing a switchgear to him and the robot making a high-quality switchgear – one that fulfills his internalized quality standards. During and after production, he checks if everything works according to his plan – which in one case needed taking over sticking bridges into the terminals from the robot. The robot is able to stick the bridges, but sometimes their positioning is slant, which does not look good and thus does not answer the worker’s expectations on a perfect product. As the worker was able to control the production process and influence the product outcome, he probably attributes the result of the work internally, feeling responsible and proud for the switchgear he produced with the robot.

### Satisfaction With the Steps of the Allocation Process

*Satisfaction* describes the matching of expectations before an action with how it is actually experienced in the situation. In the context of work there is the theory of affective events: its basis is the understanding of work satisfaction as an attitude toward work (Weiss, 2002), which results from a cognitive assessment about characteristics of the work and an affective reaction on the results of work (Weiss and Cropanzano, 1996). Affects can directly lead to behavior, but also job satisfaction can result in assessment-based behaviors (Weiss and Cropanzano, 1996).

Transferred to the process model of *ad hoc* task allocation discussed here, this means that satisfaction with the steps of the allocation process results from their cognitive and emotional assessment. There is an appraisal of the process of task allocation, the division of tasks between humans and robots (resulting allocation), the execution of the allocated subtasks and the fulfillment of the whole task (see Figure 3). The experience of one of the steps should influence the experience of the following ones as positively evaluated processes are more keen to evoke positively evaluated decisions, those allow for better work processes and therefore more satisfaction with the end results. This assessment of the whole process is then important for future behavior at work (see also Rubicon-model by Heckhausen and Heckhausen, 2018) and therefore a field important for research as well as practical application. A person who is satisfied with the allocation of tasks in interaction with a robot is more willing to work with that robot in that specific situation and probably also in different situations in the future.

Also the worker in our example feels a certain satisfaction. When asked about the allocation process, he can surely say that he is satisfied with it as he experiences process control and feels autonomous in his decisions on the allocation. He finds the robot’s criteria for self-allocating tasks appropriate and not unfair and does not have to invest incredibly high mental effort to allocate the tasks throughout the working day, experiencing support by the robot’s software on the tablet.

### Trust as an Influencing Factor

In addition to the outcome variables of task allocation, we considered it important to include also trust as an influencing factor. Trust is one of the most researched psychological constructs in human–robot interaction and also plays a crucial part in experiencing allocation processes in interaction with an automated system. Trust relates to cognitive reactions (e.g., a satisfaction judgment) as well as to the willingness to rely on the system or unit and work with them (i.e., decision acceptance), and by this it relates to behavior.

*Trust* describes the belief that an interaction partner will respect and preserve one’s interests (see e.g., Hancock et al., 2011). This belief is a basis for interacting, working and completing tasks together – not only with other human beings, but also with robots (see e.g., Rossi et al., 2017) and other automated systems - and also for accepting decisions made by the partner. Trust is especially interesting in HRI task allocation as it is unclear if people tend to separate the trust they feel for the robot they are interacting with and the trust in the support system for the allocation. As the software is closely related to the robot’s actions, it might be that there is no strong differentiation between support system and robot trust. Accordingly, Freedy et al. (2007) can show that trust in a robot affects the acceptance of information given and the willingness to follow robot-made suggestions (that are actually a result of the software/system behind). Trust also influences system effectivity and the using rate of an automated system (Lee and See, 2004) and is associated with reliance on automation (see e.g., de Vries et al., 2003).

Schaefer et al. (2016) built a model of trust in interaction with automation in general. In HRI, Hancock et al. (2011) were able to identify the attributes (e.g., type of robot) and performance characteristics (e.g., reliability) of the robot as the factors with the biggest effect on building trust. Considering the mode of automation, users tend to trust a manually adaptable automation more if it allows for explicit control (Moray et al., 2000).

The experience of the cooperation is influenced by the worker’s trust. In our example, the robot behaves carefully and the worker has not made any bad experiences with it. Hence, the worker has probably no reason to distrust the robot and the linked system for adaptively taking over tasks. It also helps that the robot is very reliable in its actions. The worker can therefore accept it as a help at work and can also accept autonomous task execution.

## Propositions – Linking the Psychological Consequences

The psychological consequences of *ad hoc* task allocation processes described in Section “Psychological Consequences of Allocation Processes” do probably not stand on their own, but are more or less tightly interconnected, as most psychological constructs are. The proposed connections are shown in Figure 3 by using (dotted) arrows and are touched on in the belonging paragraphs of Section “Psychological Consequences of Allocation Processes.” These connections between the constructs of the model represent the propositions that we make about how the different constructs relevant for perceiving task allocation in HRI are influencing each other.

These 7 propositions are:

1. All specific outcomes the model contains are influencing the satisfaction with the process step they are resulting from, except for acceptance of the allocation.
2. As explained in Section “Satisfaction With the Steps of the Allocation Process,” the satisfaction with one process step will influence the satisfaction with the next step. Hence, an iterative process with the opportunity to adapt allocation decisions is so important, as a low satisfaction with an early process step can in the end contribute to dissatisfaction with the work results.
3. Perceived autonomy influences self-efficacy and the degree to which work results are attributed internally. We believe that finding ways to generate some form of autonomy in the allocation process, even in more restricted contexts like partially automated production, is especially important as it probably has the largest effects on a number of other psychological constructs relevant for a positive experience of work and the self.
4. Trust in the system involved in the allocation in another vital influence factor for allocation process satisfaction as well as for the acceptance of an allocation made, yet it is not primarily a direct consequence of allocation process design but a prerequisite.
5. The acceptance of a resulting allocation by the worker is influenced by his satisfaction with it.
6. An allocation resulting in high perceived task identity will evoke more intense flow experiences, as goals are clearer.
7. Process control influences the mental effort the worker needs to invest in an allocation decision. This effect of a higher control perception leading to more straining of mental capacities can lead to process satisfaction being reduced because of the “burden” of more control.

Our model on *ad hoc* task allocation in HRI with the variables described and their connections (see Figure 3) shall serve as a base for research and can be used to test individual psychological outcomes of different task allocation process configurations. The propositions as well have to be tested and can be broadened and supplemented by future research.

## Discussion

The model of an *ad hoc* task allocation process and its psychological consequences presented here is the first one to broadly address a process crucial for designing cooperative work in human–robot interaction. It puts the focus not on allocation results alone or on the “optimal” distribution of tasks between humans and machines, but on the allocation process and the potential consequences it can have on employees and their cognition, emotions and behavior in cooperating with a robot. Using the concept of self-organization by including the worker in an allocation process that is integrated into the work itself opens new perspectives on how to implement HRI in a humane and efficient way.

The aim of this paper was to develop a model of the psychological effects of task allocation processes in HRI. This model is developed based on present knowledge on task allocation and psychological aspects of decision (process) perception. We want to unite insights about factors considered in optimization-based allocation models and about psychological theories and effects to help create a research base for allocation process design in worker-centered HRI. By connecting a generic view on allocation processes and experience-related psychological constructs, we develop a scientifically grounded model of psychological effects of task allocation. It can form the basis for examining the effects of different allocation processes, understanding the meaning of self-organization for HRI and motivate further research on task allocation suitable for designing humane work systems.

### Limitations of the Findings

One aspect for further developing the model is to match it to the analysis of existing and concrete practical allocation examples in manufacturing. The model we developed is derived from research insights in different fields and with different application cases, not all from human–robot or even human–automation interaction. One reason for this is that we rarely find *ad hoc* allocation processes in practical application, another one being the lack in research on task allocation from a psychological point of view. As our goal is to regard task allocation in HRI from a work psychological perspective and to help understand the mechanisms behind allocation processes, the developed model can only be a result of integrated knowledge and needs not necessarily be fully proven in reality yet. The model therefore is, just as human–robot interaction as a whole field, to be further developed and adapted to technological, societal, and other changes that shape cooperation and collaboration of humans and robots.

Another question rising is that of generalizability of the model and the propositions. The model has its root in the design of task allocation in industrial production involving the use of (collaborative) robots. It can be assumed that it does not make a big difference if the robot is collaborative or not, as long as the application range of the “classical” robot is comparable. However, often a more dynamic use is limited by cost-intensive programming of robots and due to a lack in mobility. Cases that go beyond production, e.g., service robots delivering drinks to patients in care homes, could also profit from the perspective the model offers. A caregiver being able to dynamically send the robot to patients they know enjoy interacting with it whilst freeing time for themselves to personally interact with other patients in need will probably experience similar effects, Also they will probably feel greater satisfaction due to a gain in autonomy or higher self-efficacy in executing their tasks when they know how to use the robot as a support. Essential for the generalizability is not so much the robotic appearance, being it a robot arm or a humanoid service robot, or the application context, but the opportunity to allocate tasks dynamically with relative ease during work.

### Implications for Research

The model is a first step toward research on allocation processes in human–robot interaction. It shall stimulate studies on allocation process design and will be used as a basis for upcoming experimental analyses of different configuration of allocation processes and their specific outcomes. The focus of our future research will be on the outcomes of workers’ allocation influence – as a chance to design humane, participative work that is able to fit the business expectations of efficiency and effectiveness. We hope to use our upcoming results to improve the task allocation model presented, e.g., by identifying effect sizes, and shape it toward a potential application in work design.

We regard especially the question of autonomy and opportunities for executing and experiencing control as important in designing humane HRI and would therefore encourage researchers to address these topics. What makes production workers experience autonomy in working with a robot, and how can autonomy be implied within a framework considering economical necessities? In the area of task allocation research, it is especially interesting to find the right balance to create processes that allow for autonomy while ensuring economic functioning and between not overwhelming the workers with decisions that are too complex for them and not demanding too little by not “bothering” them with involvement in robot functioning and work scheduling. For this, we need research that touches on the psychological effects of task allocation, both in laboratory and in field settings.

Research questions that we identified as central following our model are:

1. Can the proposed effects of the design of the allocation process on the psychological variables be confirmed by empirical research?
2. What are the main similarities and differences considering task allocation in HRI and allocation in human–system interaction in general or even in human–human interaction, where a lot of literature comes from? In how far are drawn conclusions valid for the use case of HRI in production?
3. Can the propositions on the connections between the psychological outcomes posed in Section “Propositions – Linking the Psychological Consequences” be verified by laboratory and/or field experiments on task allocation in HRI?
4. Does a shared influence over task allocation of worker and robot lead to the same positive effects as an allocation that is only influenced by the worker?
5. How can a system for task allocation be designed to allow for system transparency and process control with the worker that as well limits the mental effort needed to interact with it?

Finally, further aspects of interest are the effects of the social perception of the robot on task allocation and HRI. Just as how much a person trusts a robot, it is important how the social system of a workplace is and how it is perceived by the workers involved. How do they see their roles, what other people and social roles are involved and what is the robot to them: Is it perceived as a teammate, a tool or the “robot boss” (for research on this, see e.g., Rossi et al., 2019)? Future research should focus on the implications such perceptions of the robot and associated self-perceptions can have on how tasks are allocated, which allocation processes are preferred under which conditions and how task allocation can contribute to a configuration of the social system that is desirable (e.g., in satisfying the need for relatedness, see Ryan and Deci, 2000).

### Implications for System Design in HRI

The focus on the human in designing HRI, and specifically in approaching task allocation, does not only lead to the direct effects considered in this model, but also has long-term effects on wellbeing, health and employability (for a review on ergonomic design of human–machine interaction and psychological wellbeing see Robelski and Wischniewski, 2018). A short-term dissatisfaction with an allocation solution can lead to ongoing dissatisfaction with the way tasks are distributed and the unwillingness to cooperate with a robot that “always gets the better tasks.” On the other hand, a well-designed allocation process that includes workers can empower them to make their own decisions and can help to involve them in task planning and interaction with technology. In this way, employees can develop, handle new challenges better and maintain their mental and physical health, as they can adapt to changing demands. It can contribute to reaching the ideal of a real differential work design that takes into account the individual needs and premises of every worker. This should not only be the goal of work psychologists, but of everyone designing and applying HRI systems.

Although the model is a first step in considering processes and their psychological effects in allocating tasks in HRI, there are still points to consider from a practical point of view: first and foremost, one can see the importance not only of designing the technology and the tasks but also of considering the allocation process as a designable feature of work. Although it is partially preparatory, it is still of vital importance for the experience of work and human behavior beyond the allocation process itself.

A first design principle that arises from the research presented is the participation of workers in the allocation process. The importance of autonomy and control for creating accepted allocation processes and by this acceptable interaction with a robot shines through in most aspects considered. It makes people feel self-efficacious and able and willing to handle daily hassles. Concretely, this means that existing and new tasks have to be analyzed thoroughly to identify the necessary steps for their fulfillment, the affordances of each step and the capabilities of all agents, human workers, robots, and other machines involved. One result of this task analysis is knowledge about leeway in executing the subtasks. After considering factors that limit that leeway to some extent (e.g., a high ROI of a robot), the remaining opportunities are then not condensed to one fixed solution of task allocation, but are opened up to the worker to autonomously decide on task allocation and to take part in designing their personal HRI.

A second design principle is the use of more self-organization in task allocation instead of pre-planning. Leaving the authority over task allocation to the human–robot team, and allowing for a combination of human situation analysis and expertise and the technological opportunities of adaptive behavior and execution reliability can contribute to realizing the ideas of industry 4.0 whilst considering human needs and success factors for human–robot interaction. On the robot’s side, the application of self-organization needs at least tractability by the worker and flexibility in using the robot for a number of different tasks without complex retooling processes in between. In addition to this, implementing some form of adaptivity as in the example, i.e., using sensors to track the production status and human actions and develop these information autonomously into appropriate actions, can make task allocation a much more interactive process where the worker can share allocation authority. This can help creating a fluid production process, remove strain from the workers when necessary, enhance efficiency and help create a feeling of team effort toward a task. This self-organizational approach in general needs clear boundaries for the system, i.e., clearly and understandably communicated borders for what is possible and what is not, to ensure efficiency and a fluid integration into other work processes. To work out those boundaries without limiting the system’s power to adapt dynamically is one big challenge for practitioners.

The third design principle is to create a process that offers all the information necessary for a (partially) automated allocation decision (as in the case of adaptive robotic behavior) to the workers involved. A transparent process that is accountable, based on acceptable and understandable decision criteria that are communicated to the workers in a form that does not overexert them but gives them just the right amount of information, is key to designing good HRI. Designing a user interface that delivers all needed information in a user-friendly manner is a comparably easy but very promising way to achieve higher trust and acceptance and thus create a satisfying work environment for employees working with robots.

All of these steps require appropriate qualification of the worker before the implementation phase. Preparing workers for understanding automated decision-making, principles of adaptivity and the criteria behind allocation decisions and teaching them how to intervene if necessary is a vital step in the change process of implementing HRI and new forms of task allocation. Also, their feedback in this phase can then be used to improve the processes before they are implemented. This allows for an accepted and clear framework for self-organized *ad hoc* allocation in HRI providing the necessary borders and freedoms in allocating tasks between workers and robots.

For now, the *ad hoc* allocation model and the concepts behind can inspire practitioners to think about alternative ways of allocating tasks than to just pre-plan processes and then leave them unchanged until they are no longer needed. *Ad hoc* allocation that can dynamically adapt to changing conditions can unlock new opportunities for HRI and for empowering workers to influence their own work design.

### Conclusion

In general, a psychological view on task allocation in HRI is lacking in research and we need a deeper examination of different steps and aspects of *ad hoc* task allocation to be able to design not only efficient, but also humane human–robot interaction. The model presented here might be a first step, but is open toward further development, new insights and extensions broadening the picture of the influence of task allocation processes on humans.

## Data Availability Statement

The original contributions presented in the study are included in the article/supplementary material, further inquiries can be directed to the corresponding author/s.

## Author Contributions

This paper was conceptualized and written by the corresponding author AT. LA and AK were revising it critically multiple times during the writing process. All authors approved the submitted version.

## Conflict of Interest

The authors declare that the research was conducted in the absence of any commercial or financial relationships that could be construed as a potential conflict of interest.
